# Efficient 3D Objects Recognition Using Multifoveated Point Clouds

**DOI:** 10.3390/s18072302

**Published:** 2018-07-16

**Authors:** Fabio F. Oliveira, Anderson A. S. Souza, Marcelo A. C. Fernandes, Rafael B. Gomes, Luiz M. G. Goncalves

**Affiliations:** 1 Department of Computer Engineering and Automation, Federal University of Rio Grande do Norte, Natal, RN 59.078-970, Brazil; fabio.veritate@gmail.com (F.F.O.); mfernandes@dca.ufrn.br (M.A.C.F.); rafaelbg@dimap.ufrn.br (R.B.G.); 2 Department of Computer Science, State University of Rio Grande do Norte, Natal, RN, 59104-200 Brazil; and.abner@gmail.com

**Keywords:** multifoveated structure, 3D object recognition, point clouds

## Abstract

Technological innovations in the hardware of RGB-D sensors have allowed the acquisition of 3D point clouds in real time. Consequently, various applications have arisen related to the 3D world, which are receiving increasing attention from researchers. Nevertheless, one of the main problems that remains is the demand for computationally intensive processing that required optimized approaches to deal with 3D vision modeling, especially when it is necessary to perform tasks in real time. A previously proposed multi-resolution 3D model known as foveated point clouds can be a possible solution to this problem. Nevertheless, this is a model limited to a single foveated structure with context dependent mobility. In this work, we propose a new solution for data reduction and feature detection using multifoveation in the point cloud. Nonetheless, the application of several foveated structures results in a considerable increase of processing since there are intersections between regions of distinct structures, which are processed multiple times. Towards solving this problem, the current proposal brings an approach that avoids the processing of redundant regions, which results in even more reduced processing time. Such approach can be used to identify objects in 3D point clouds, one of the key tasks for real-time applications as robotics vision, with efficient synchronization allowing the validation of the model and verification of its applicability in the context of computer vision. Experimental results demonstrate a performance gain of at least 27.21% in processing time while retaining the main features of the original, and maintaining the recognition quality rate in comparison with state-of-the-art 3D object recognition methods.

## 1. Introduction

With technological advances experimented in hardware, artificial vision systems can now capture and process real-world depth data in addition to color information. The use of these data, inherent to the 3D space, becomes an interesting alternative for execution of tasks in robotic vision, in real time. This is the basis of the system developed in this work that deals with the real-time capture and interpretation of data in three-dimensional format, since they offer more details in the abstracted information. Actually, most robotic systems based on computer vision have sensors based on 2D images. However, there are some types of robotic applications that require information about observer-to-scene distance (i.e., depth) for proper performance of some tasks. Nevertheless, the development of artificial vision applied to robotics is hard, and the retrieval and efficient use of three-dimensional information is still a challenge.

Hardware evolution has led to the development and dissemination of RGBD devices as the Intel RealSense SR300 [1] or the Microsoft Kinect [2], which evaluates 3D world coordinates (D) of scene points and captures red (R), green (G), and blue (B) intensity values (or RGB data) at these 3D points. Other devices such as the ZED camera [3] or the Bumblebee camera [4] have also allowed the real-time acquisition of 3D points with associated RGB values (or point cloud, as known). Thus, the input data model used in this paper is the 3D point cloud (PC) that provides high amount of scene information observed from the acquisition of the PC by any RGBD device, such as the above ones.

The advantages of point cloud over 2D images and the interest to extend the approach of multi-resolution with Moving Fovea (MMF) [5,6] has led Gomes et al. to import the characteristics of MMF into 3D point cloud resulting in a model that they called *Foveated Point Cloud* (FPC) [7]. In fact, in the paper on *Efficient 3D Object Recognition Using Foveated Point Clouds*, Gomes et al. [7] use a foveated structure (FS) to perform the detection of objects in a scene represented by a point cloud, to decrease the number of data during the processing. That work shows that this reduction brings benefits in the performance of some task being executed, which takes less time without necessarily affecting the results precision. Therefore, the main purpose and the proposal of this paper is to extend the model based on the FPC [7] coming up with a sampling approach to 3D data that reduces processing time while achieving multiple foveated point clouds (MFPC) at several points of interest. This extension allows using this approach to deal with, for example, the search of multiple objects in a scene instead of a single one as in the previous work.

The contribution of this work is the approach to speed-up the processing on multifoveated point clouds by avoiding redundant computations in overlapping regions. The novelty in relation to previous work is that, instead of only one moving fovea, several can be used at a time without redundancy on the computations. The results of the experiments provided in this work show that this reduction brings benefits in the performance of some tasks being executed, such as recognition, speeding up the applications without affecting the precision. This makes possible the use of multifoveation to deal with the search of multiple objects in a scene, instead of just one object as in previous work [7]. Consequently, multiple targets in a scene can be addressed in robotics applications. Other visioned applications as 3D data transmission through the Internet where data reduction is mandatory can also use such approach.

Thus, the scope of the present model is restricted to visual data features capture with depth data using several types of structures in parallel, which reduces the amount of information without creating redundant information to streamline a task in dynamic or static environments.

This article is structured as follows: Section 2 presents the theoretical background used in this work with revisions of some related works that deal with the recovering of 3D objects, multi-resolution and multifoveated approaches. Section 3 describes the application of multiple 3D moving foveae in the problem of recognition of various objects and their formulation in the context of our proposal. Section 4 describes the experiments, including illustrations and scene and model data. Section 5 describes the results a performance evaluation from a dataset, while Section 6 closes the article with our final remarks.

## 2. Background Theory

To achieve high level of autonomy, it is necessary for a robot to react without human intervention to stimuli provided by the environment [8,9,10]. Robotic vision applications use several types of sensors to capture stimuli data. For example, the above mentioned types (Bumblebee, Kinect, ZED, or Intel RealSense SR300) are being used nowadays in robotic vision, often known as depth sensors. To better comprehend the approach to multifoveation in point cloud proposed in this paper that can be used for robotic vision, in this section, we present the basics of multi-resolution and the previous foveation approaches, starting with the single foveation that has allowed performing real-time tasks mainly for robotic vision. Further, some brief background is discussed about object recognition that is not trivial if using 2D images and is even more complex in 3D data approaches.

### 2.1. Multiresolution and Foveation

To make the right decisions in robotics, one must process all of the relevant data available. In general, it is not possible to process such huge amount of data at one available time slot. However, in a dynamic and cluttered world, it is rather important to filter information. Moreover, dealing with visual information with just a single focused look is not enough to perform general tasks. Our brain can acquire and abstract visual information quickly and efficiently deciding where to focus and what is the sequence of fixations [11]. This sequence of fixations is related to cognitive mechanisms controlled by our visual attention mechanism. It is understood that the biological system of human vision has two types of visual attention behavior called top-down and bottom-up [12,13]. Top-down attention is an approach that refers to the internal orientation of care based on prior knowledge, desired goals, and planning. In contrast, bottom-up attention is directed purely by stimuli of external factors that stand out because of their inherent properties in relation to the background.

**Figure 1:**
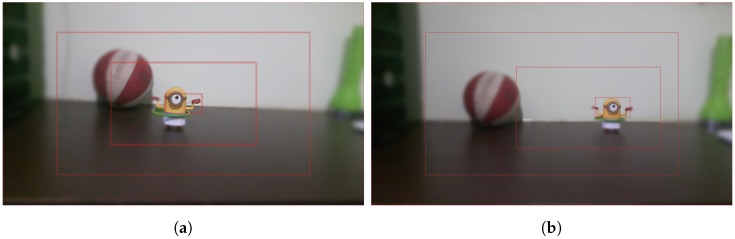
Representation of the application of the foveation in multi-resolution: with centered fovea (MCF) [13] (**a**); and with moving fovea (MMF) [5] (**b**).

Thus, no matter which of the above tasks is performed, they need high processing rates, which makes vision one of the most complex activities for a robotic system implementation, since a single view requires a huge amount of data. Thus, using some technique for diminishing the amount of input data has proven to be a good idea [5,13]. Many types of methods optimize image processing tasks. One of these is the representation based on the non-uniform density called foveation, which has as one of its aspects a biologically inspired model that mimics the mapping of the retina to the visual cortex to deal with this amount of data [14,15,16,17,18]. Foveation aims to work in such a way that can be considered similar to the retina, which has the specialty in maximum visual acuity at the fovea, as noted in Figure 1a,b. Figure 1 shows such a scheme explaining the model based on foveation. It has a high resolution in some smallest area, or volume in our case, of the data structure, called the fovea, and decreases resolution according to the distance from that high resolution zone.

The foveation process can be performed by means of software downsampling [14], with reduced sampling [19] for systems that use at least two cameras [20,21]. Each type of execution of the foveation process has its relevant advantages and disadvantages. Foveation through software allows for easier changes, facilities, and flexibility to be implemented in conventional hardware, although it is slower than hardware solution which are generally much more expensive and difficult to change. In this article, the foveation technique is implemented in software.

### 2.2. Multifoveation

In some cases, it is possible and often necessary to have several foveae spatially distributed in a data space, each one covering one small region of the data; this is called multifoveation. The use of multifoveation is the case, for example, in robotics vision when a robot wishes to track multiple targets. Notice that multifoveation can be straight implemented in several contexts through the repetition of several foveated structures.

Nevertheless, the simple multiplication of the foveated structures in the system cause intersections between them and, consequently, produces redundancy in the analysis. The articles found with this problematic are the ones done by Camacho et al. [22] and Rodriguez et al. [23]. Regions of interest are encoded as a special case of elements belonging to more than one region within the foveated structures. For these approaches, areas of interest are encoded as a special case of pixels belonging to more than one region in the union of the structures. The highest resolution levels (i.e., the fovea regions) are processed separately from the FS to minimize the redundancy processing generated by the structures encounters. However, this approach is only an attenuation because there are redundancies of the structures in the other levels that have intersections, which are not treated in those works.

### 2.3. A Note on 3D Data Recognition

Traditionally, object recognition systems obtain depth data from costly and rarely available range sensors such as laser scanners [24,25] and structured light patterns [26]. The high processing required until last decade for 3D object recognition systems has caused them to be performed off-line [27]. Although they are in constant evolution since they are modified as new algorithms are proposed by the robotics community [28,29].

In recent years, algorithms designed to describe 3D surfaces through signature and histograms of local geometric traits that have had better performance, accuracy, and reliability [30] compared to using only one of these [28,31,32,33]. 3D object recognition systems have been developed based on the correspondence of key-points and descriptors extracted from the point cloud of the scene and object sought. The correspondences of the points are grouped by hypotheses that share a common transformation, based on voting [34,35], multi-dimensional grouping [36,37] or RANSAC [38]. Thus, to identify the object, a reliable method is needed to deduce certain conditions, such as the number of votes, the size of the group, or the number of inliers in RANSAC.

## 3. Related Works

The relevant contributions of multifoveation to the pattern recognition, computer vision, robotics and computer graphics communities are briefly addressed here, being discussed mainly with focus on the methodologies for data acquisition that are employed in each of these areas. The advantages of the method proposed in this article are enhanced in respect to those related works, where applicable.

There are works in robotic vision field related to reducing the amount of data to be encoded/decoded [7,13] and also in foveated systems for real-time video transmission [15,17,23]. In these applications, data are encoded with foveation, thus maintaining higher resolution in the areas of observation. Basu et al. [39] proposed a 3D vision system with limited bandwidth throttling, where the texture quality and the resolution of objects are controlled by the position of the fovea. To keep efficiency while diminishing the execution time, Beserra Gomes et al. [7] used a foveated system to identify objects in (3D) clouds of points in real time, where the fovea position tries to reach the desired object. A similar approach is also applied to 2D images for tracking and object detection [6].

Among the works on multifoveation, we found several that perform the extraction of movement resources to infer knowledge about the context, for example, the ones of Dhavale and Itti [40], Camacho et al. [22] and Rodrígez et al. [23]. From these, there are some software capable of performing real-time multifoveated computing, but for this they use dedicated reprogrammable hardwares, such as FPGAs [22] which have excellent parallel processing capability. There are also multifoveated algorithms in the reconstruction of 2D images, such as the one by Tabernero et al. [41]. There are works developed in software, using 2D images, dealing with multifoveation, such as the one of Medeiros [42], which developed such a similar approach for construction of a 2D multifoveated structure from input images that makes easy feature extraction.

The increasing availability of cheaper and more effective 3D sensors such as the Microsoft Kinect has helped in the publication of various works on the recognition of 3D objects approaching this class of sensor [29,43,44,45,46,47] Clustered Viewpoint Feature Histogram (CVFH) was proposed by Aldoma et al. [29] with a global feature to improve the performance of object recognition in robotics. Ashbrook et al. [24] introduced an approach that allows the recognition of objects in a robust and efficient way in two dimensions and can represent and classify the form of arbitrary surfaces in three dimensions extracted from 3D data and with the Hough transform [48]. The Point Clouds Library (PCL) can be used to shape 3D object recognition systems and evaluate estimation based on local and global resources [43]. Aldoma et al. [43] implemented an approach based in the execution of the algorithms within the PCL framework.

We notice that only a few of the above works treats foveation in the context of point cloud [7] and a few others treats information redundancy in foveated images [22,23,42]. No work was found doing both at the same time. This reinforces the need for this type of approach for treating PC and verifies the novelty of this work.

## 4. Multifoveated Point Cloud-Proposal

Our proposal in this paper is based in the FPC [7] with the ideas of models of multifoveation 2D [22,23,40,41]. By grouping these two bases, it is proposed to create a model that reduces the processing time in the detection of objects, avoiding the processing of redundant regions generated by the overlap of the foveated structures, allowing a better performance in a multiple objects recognition task.

The idea is to apply several foveated structures on the PC, but with an additional step that remove the redundant points. In this scheme, it is possible to reduce the processing time while the densities around the several foveae are sufficient to guarantee the detection of the objects. In this scheme, the FS is a sectioned frame having different resolutions (multi-resolution), where the size of the level is inversely proportional to the quality of the resolution, so that each of these levels is successively encompassed by the other in such a way that all are disjoint.

### 4.1. Approach to Cloud Multifoveation

The FPC proposal has as the main mechanism the downsampling in the original point cloud using concentric boxes, where each one represents a level, producing a PC with a different density for each box. In the context of multiple foveated structures, applying multiple moving foveae would result in a considerable increase in processing, since intersections between regions of distinct structures would be processed several times. In this way, our approach to the multifoveated model has as a specific case the proposal in the previous FPC. The foveated point cloud is achieved by downsampling the original PC using concentric boxes, each representing a level, which is the case for only one foveated structure. The operation of multiple structures without treatment is similar, although redundancy is eventually generated in regions to be processed, in addition to having more points than in the original scene.

### 4.2. Intersection Regions between Levels

The proposed multifoveated point cloud is composed by *n* structures with the same number of levels. However, adding several identical points in the PC is allowed by the storage data structure of the PC because it is basically a list of points and not pixels that have their values set on the display device with only one value at a time. To simplify this aspect of the PC, we name as the computational space (virtual) the representation of the 3D virtual region that has the peculiarity of allowing the existence of several identical points in the scene. Let (x,y,z) the world coordinate system of this computational space. However, the amount of points is limited to the available memory of the operating system to which the file belongs. Nonetheless, the *raster images* are already limited by a standard format of the acquisition device or by the display device.

We are aided by set theory area to describe and determine the intersections between regions. Before performing this analysis, we define four specific sets: $\chi_{v}\left(i,j\right)$, $B_{i,j}$, U and *R*. The operating ranges (i.e., zones of possible modifications) of the FS levels in the 3D computational space are defined by $\chi_{v}\left(i,j\right)$ according to Equation (1), where i∈[0,1,…,m] and j∈[1,2,…,n]. Term *v* is one of the components of (x,y,z). The action stretches χ(i,j) for each component of the parallelepiped relative to the level *i* of a foveated structure *j* is defined according to Equation (1):

(1) $$\begin{array}{c} \chi_{v}\left(i,j\right)=v_{j}+\delta v_{i,j}\leq v\leq v_{j}+\delta v_{i,j}+Sv_{i,j} \end{array}$$

∀v∈(x,y,z) and $\chi_{v}\left(i,j\right)\in R$. The terms ${\mathrm{ffi}}_{k}$ and $S_{k}$ are the displacement and size, respectively, as defined in the work of Beserra Gomes et al. [7]. They are given in function of the level *k* by ${\mathrm{ffi}}_{k}$ = $\left(\delta_{x},\delta_{y},\delta_{z}\right)$ and $S_{k}$ = $\left(S_{x},S_{y},S_{z}\right)$.

Each of the levels determines a subset of $R^{3}$ defined by $B_{i,j}$ according to Equation (2). Regions $B_{i,j}$ are such that they obey Equation (3) to *f* constant, variation of *i* levels and any foveated structure. They are composed of their respective intervals in the Cartesian axes, which are expressed by $\chi_{v}\left(i,j\right)$ (Equation (1)) which defines the performance sections of each region, when observed the three axes (x,y,z) from the full volume of the related level.

(2) $$B_{i,j}=\{\begin{array}{l} \left\{ \begin{array}{l} \begin{array}{c} \left(x,y,z\right)\in R^{3}\;|\;\left[\chi_{x}\left(i,j\right),\chi_{y}\left(i,j\right),\chi_{z}\left(i,j\right)\right]\\ \wedge \;\left(x,y,z\right)\notin \overset{m}{\underset{k=i+1}{⋃}}B_{k,j} \end{array}\;i<m \end{array}\right.\\ \left\{\left(x,y,z\right)\in R^{3}\;|\;\left[\chi_{x}\left(i,j\right),\chi_{y}\left(i,j\right),\chi_{z}\left(i,j\right)\right]\right\}\;i=m \end{array}$$

The definition of 3D foveated structure is given by Equation (4), also for *f* constant, being able to admit the values of *j*.

(3) $$\begin{array}{c} \overset{m}{\underset{i=0}{⋂}}B_{i,f}=\emptyset \end{array}$$

(4) $$\begin{array}{c} U_{f}=\overset{m}{\underset{i=0}{⋃}}B_{i,f} \end{array}$$

We define U as the set formed by finite foveated structures, where each of them contains a finite number of levels, according to Equation (5).

(5) $$\begin{array}{c} U=\overset{n}{\underset{j=1}{⋃}}U_{j}=\overset{n}{\underset{j=1}{⋃}}\overset{m}{\underset{i=0}{⋃}}B_{i,j} \end{array}$$

In the MFPC model, for a given level *i* of the FS *j*, the density reduction factor $d_{i}$ is constant for all space $B_{i,j}$ with each having its estimated density [7]. In the multifoveation context, when two or more sections of different indexes of levels intersect, there will be regions for which different reduction factors will be applied. In this case, these structures should always have the same number of levels. So our proposal is defined only in the case of multiple FS that have the same number of levels. The density question is important because it defines how the addition of the points in the structures will be evaluated.

A priori the homogeneity of some region of a FS means that the region does not have intersection with higher levels (a level *i* is higher than the level *j* case i>j and lower case i<j) or has no intersection at all, that is, the entire region of $B_{i,j}$ has a density reduction equal to or less relative to that level, considering that the density of the region is already less than calculated it would not be possible to establish the density defined by the level (e.g., a region without points).

**Figure 2:**
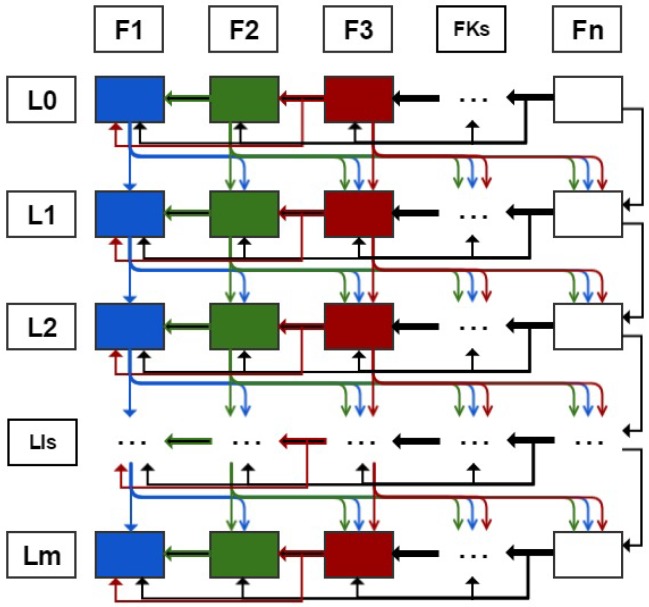
Representation of the redundancy validation operation diagram in 3D multifoveated point cloud. The structures shown in the columns and enumerated from F1→Fn and their respective levels have been organized into enumerated lines of L0→Lm. The direction of the arrow indicates the start and finish of the point check.

The redundancy validation operation diagram in the 3D multifoveated is shown in Figure 2. It has its scheme divided into *n* FS and m+1 levels. The checks of a point of the non-zero PC that belong to any element of U are made by the directions of the arrows. Thus, it is verified whether a point *p* belongs to the same level of previous structures or to a higher level of successor structures. If it is affirmative the point is not added to the level of the foveated structure observed $B_{i,j}$, otherwise it will be added. This diagram is the representation of how the redundancy treatment algorithm of this proposal works. For each point, the computational complexity in both the verification of the same predecessor levels and the successors at higher levels is O(n).

The operation of the redundancy treatment is given by the choice of a generic point *p* non-zero of $B_{i,j}$ (or region *R*) in the PC. Given that we are observing a foveated structure $F_{j}$ on one level $L_{i}$ (box $B_{i,j}$) two searches are done. The first is made on the same level $L_{k}$ as the predecessors of the structure (structures that were created earlier $F_{1}\to F_{j-1}$), analyzing whether the observed point belonged to any of these previous levels. The second search is done on the $L_{i+1}$ of successor FS (structures which will be created from $F_{j}\to F_{n}$), given that the observed point belongs to some of these higher levels of successor FSs. If neither of these two tests is positive, then the *p* (or region analyzed *R*) will be added in $B_{i,j}$ (the box analyzed), which is basically the function of adding points $A_{p}\left(B_{i,j}\right)$ made explicit in Equation (6).

(6) $$A_{p}\left(B_{i,j}\right)=\{\begin{array}{l} \;1\;\;\mathrm{if}\;\;p\notin (U-B_{i,j})\\ \;1\;\;\mathrm{if}\;\;p\notin \left(\overset{i-1}{\underset{f=1}{⋃}}B_{k,f}\right)\;\wedge \;p\notin \left(\overset{n}{\underset{f=i}{⋃}}B_{k+1,f}\right)\\ \;0\;\;\mathrm{if}\;\;p\in \left(\overset{i-1}{\underset{f=1}{⋃}}B_{k,f}\right)\;\vee \;p\in \left(\overset{n}{\underset{f=i}{⋃}}B_{k+1,f}\right) \end{array}$$

From the development of Equation (6), a method has been implemented that allows the process of selection of points and *features* to be sampled in each level of the FSs, removing the points and *features* that generate ambiguity, deformations in the scene and, consequently, errors in the integrated tasks with our mechanism. Its operation is illustrated in Figure 2; we can suggest a verification situation of a point that is in the second FS in level 1 (i.e., in $F_{2}$ and $L_{1}$), so the checks would be performed: first, on the levels L2 of the second fovea on (i.e., $F_{2}\;to\;F_{n}$ green arrows); and, after that, the checks would be made in the levels $L_{1}$ of the FSs previous to $F_{2}$, that is, only in the FS $F_{1}$, so which the point validation would be done from the cases of Equation (6). This whole procedure depends on the point to be checked and where it is embedded in the example, in FS $F_{2}$ and level $L_{1}$.

**Figure 3:**
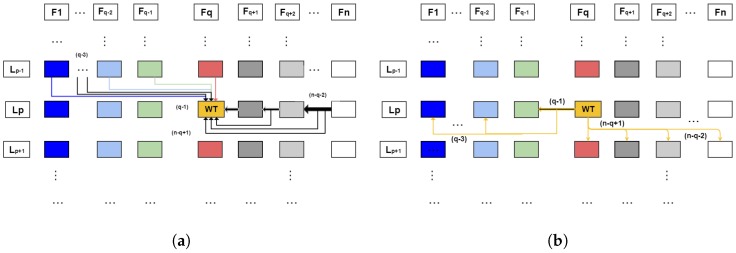
Representation of the redundancy validation operation diagram. We organized the foveated structures seen in the columns and respective levels rows: (**a**) the search carried out in WT; and (**b**) the search processed by WT, considering WT belongs to a general foveated structure and level in the system.

For clarity, in Figure 3a,b, we establish a level p in a foveated structure q called WT shown in yellow. They are an observation of how the mechanism works given a separate observation. In Figure 3a, the search process on WT is shown where the validation is made by the predecessor levels p−1 to the foveated structure q (i.e., current level and levels already searched). The search is performed at the same level of structure from p+1 to n. In Figure 3b, we have the search path made of WT which are *backward* and *forward*, so the WT searches are done at the same level as its $L_{p}$ and for all previous structures being searched *backward* observes if the point has already been inserted in previous levels. The search *forward* is done to know if the point belongs to a level with greater magnitude of density than WT, which is made in the $L_{p+1}$ and posterior structures. The search is done only up to p+1 levels, since the $L_{p+1}$ covers up to the $L_{m}$.

### 4.3. Multifoveation Point Clouds Model

In the multifoveated approach, each parameter becomes a set of specifications. Thus, we have that the size of these sets of parameters depends on the amount of foveated structures arbitrated in the system, as seen in Equation (5). We get the specs sets for the multifoveated point clouds model that belong to a foveated structure *l*.

(7) $$\begin{array}{c} {\mathrm{ffi}}_{k,l}=k\frac{S_{m,l}+S_{0,f}+2F_{l}}{2m} \end{array}$$

Notice that ${\mathrm{ffi}}_{k,l}=\left(\delta x_{k,l},\delta y_{k,l},\delta z_{k,l}\right)$ and $S_{k,l}=\left(Sx_{k,l},Sy_{k,l},Sz_{k,l}\right)$ shall be subject to the same restrictions as in ${\mathrm{ffi}}_{k}$ e $S_{k}$ of MFPC, respectively.

(8) $$\begin{array}{c} S_{k,l}=\frac{k\left(S_{m,l}-S_{0,l}\right)+mS_{0,l}}{m} \end{array}$$

This process of adaptation is chosen for the other equations of the foveated point clouds model [7].

### 4.4. Downsampling

Downsampling is the main step to make this model characteristically foveated because it brings multi-resolution, which is one of the attributes of the foveation model. Thus, we decided to adopt the same downsampling system used in the FPC. After calculating and defining the level limits of each structure, the point cloud is sub-sampled to change the density of the PC (i.e., change of resolution). There are two possibilities for storage of the PC [7]: (1) create a single PC that gets together all sub-sampled points; and (2) store, independently, each PC that corresponds to each level of the sub-sampled foveated structure. Notice that both could be applied simultaneously.

Geometric distortions are generated by joining all points in a single cloud, which is imperceptible in a visual inspection if the downsampling algorithm modifies the coordinates of the points. Thus, the solution of the problem is to group all the points of a level that do not belong to an internal, since all levels of the structures are hollow except the fovea boxes. In this way, the points of one level do not mix with points of another, keeping the restriction referring to Equation (3). To ensure the disjunction between levels, just test if the point of a *k* level to be inserted in the cloud is not inside the highest-level box k+1 to (k≠m) as seen in Algorithm 1.
**Algorithm 1:** Processing steps applied to the MFPC.
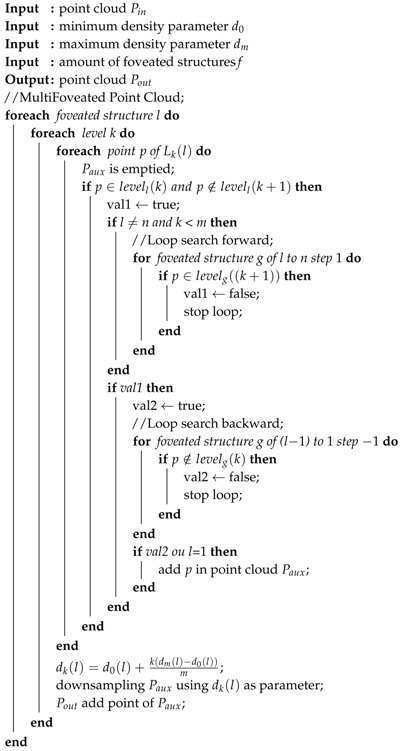


## 5. Proposed Object Recognition Scheme

**Figure 4:**
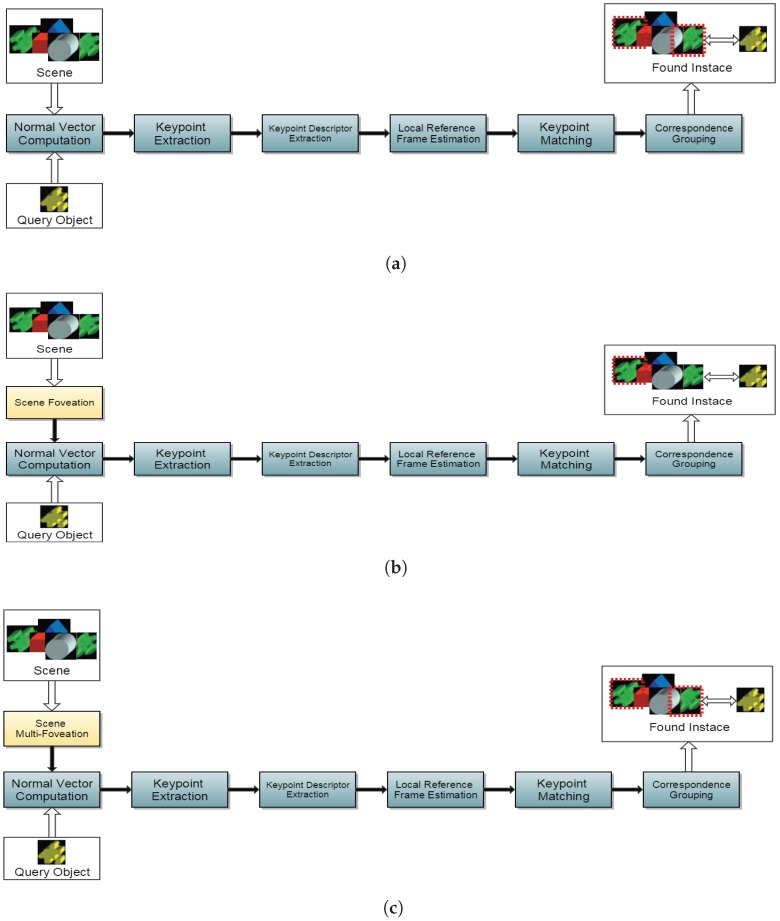
The 3D object recognition algorithm based on matching: (**a**) the original scheme [35]; (**b**) the FPC scheme [7]; and (**c**) the proposal with the object recognition scheme 3D multifoveation. In (**b**), the scene is downsampled through foveation, considerably reducing the number of points to be processed without compromising overall accuracy. In (**c**), the scene is downsampled through several foveated structures in a similar way to (**b**), as described in the text.

In this section, we discuss the application of the proposed approach coming up with a new correspondence grouping algorithm, which is somewhat similar as the one presented by Tombari and Stefano [35]. The traditional flow structure as proposed by Tombari and Stefano [35] is presented at Figure 4a. Previous work scheme of Beserra Gomes et al. [7] is shown at Figure 4b and the approach that is proposed in this work is introduced in Figure 4c. The search is done only in the object-to-scene direction and not the scene to the object, allowing the system to find multiple instances of the same object in a single scene. 

### 5.1. Extracting Local 3D Descriptors

First, the correspondence grouping algorithm allows performing a description in both the scene and the object model in PC. Thus, we obtain the direction of the normal of each point (local descriptor 3D) by estimation considering a linear variety (surface) generated by a neighborhood of size $k_{n}$ around each point. Then, a uniform downsampling algorithm is applied to extract the key-points as the centroid of all points contained within a radius $r_{k}$. After this, 3D SHOT descriptors are computed, assembling a histogram of normals within a neighborhood of radius $r_{s}$ as the signature of a key-point. The estimation of an LRF for model and scene key-points is the last step.

The algorithm by Petrelli and Stefano [49] is used here to compute the position of the key-points. Since it makes the robust estimation for each position of the key-points within a neighborhood of radius $r_{l}$ in the main axes covering an LRF. The result of this calculation generates three-unit vectors for each main direction associated with each key-point and is used in the final phase of the object recognition scheme. Different values for the parameters are defined in the scene and object model, allowing for more precise recognition tasks. Thus, the process that extracts the descriptors for the object model and the scene is shown in Algorithms 2 and 3, respectively, with parameters $k_{nm},\;r_{km},\;r_{sm},\;r_{lm}$ used for the object model and $k_{ns},\;r_{ks},\;r_{ss},\;r_{ls}$ used for the scene, respectively, the normal k-neighbors, key-point radius, SHOT descriptor radius and LRF radius.
**Algorithm 2:** Processing steps applied to the model point cloud.
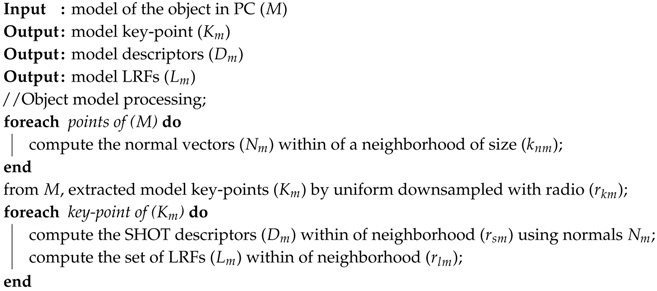

**Algorithm 3:** Processing steps applied to the scene point cloud.
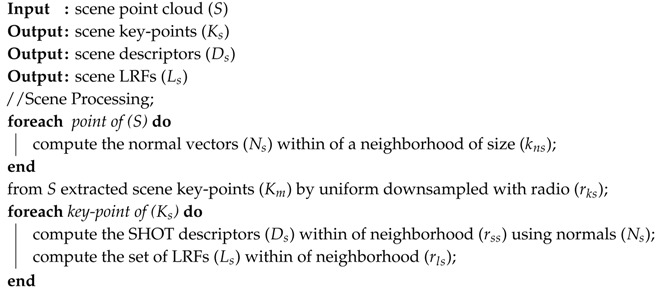


### 5.2. Keypoint Matching

In the correspondence grouping, an efficient technique was proposed by Tombari and Stefano [35]. Thus, for correspondence, there is a respective descriptor associated with a key-point and LRF for the point-to-point determination between model descriptors and those of the scene. In the model the points corresponding to the scene are searched, so that the most similar points of the scene are searched for in relation to the model. These matches are located based on the descriptors of the most similar model relative to the Euclidean distance from the n-dimensional space that the SHOT descriptors contain. The search structure used is defined based on a kd-tree [50] to deal with the heavy computational routines involved. Thus, a point matching is established when the quadratic distance between the SHOT descriptors is smaller than a threshold $d_{max}^{2}$, where this relationship process is best highlighted in Algorithm 4.
**Algorithm 4:** Keypoints matching.
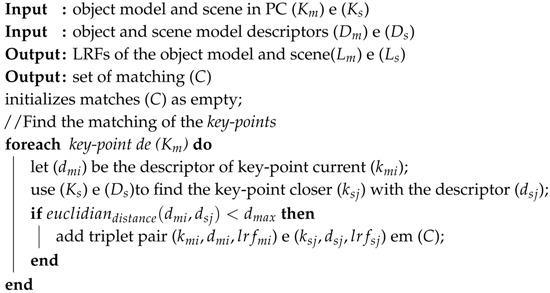


### 5.3. Correspondence Grouping

In the object and scene model, each key-point has an associated LRF, so it can perform a complete transformation of the rigid body that is modeled by rotation and translation. According to Tombari and Stefano [35], an estimate can be calculated between associated LRFs in each key-point match; consequently, a 3D Hough Space is used to gather evidence of the presence of the object through a voting process. Next, the rigid body transformation is applied, so that the Hough space cell containing this 3D position is calculated and its accumulator is incremented. Finally, after the above steps are performed for each match, the instances of the objects are found by considering each cell. From the number of votes greater than an arbitrary threshold $V_{h}$, the size of each cell is weighted by a parameter $L_{h}$. Algorithm 5 illustrates the object recognition scheme by means of matching by using 3D Hough Space to find objects in 3D space.
**Algorithm 5:** Object recognition based on correspondence grouping.
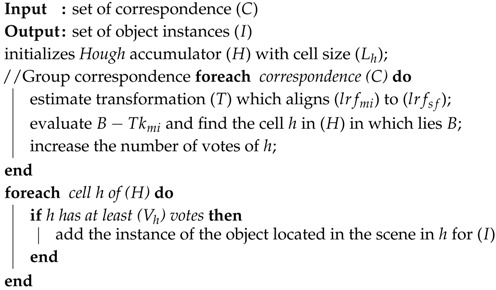


### 5.4. Object Recognition in Multifoveated Point Clouds

The FPC algorithm [7] has been implemented to improve the capabilities of 3D object recognition in the correspondence grouping approach. However, there was a problem accentuated with the descriptor used at that time. In the SHOT descriptors [30], the point density variation was the most challenging annoyance described, causing considerable problems for the execution of methods that depended on it.

Since the foveated point clouds algorithm is based on the multi-resolution that causes the variation in the density of the set of points, it was empirically noted the need to develop a descriptor that has an invariance appropriate to the fluctuation in the density of points. Because the descriptor does not have the necessary robustness for the variation of the density of points, this brought the problem of not recognizing objects when it belonged to more than one level of the foveated structure as verified in [7]. This problem no longer exists in the SHOT descriptor with the changes that brought a greater invariance to the density of points, according to Salti et al. [30].

The MFPC is applied to according to Algorithm 1 and the parameters described in Table 1. The estimation of the normal of each point can be made previous or after the foveation process. When choosing to make the estimation previously, computation is costlier, but the captured geometric traits of the scene are less distorted. However, we continue to choose to preserve the geometry of the scene, prioritizing the best accuracy.

To respect the multi-resolution of the scene PC, we use key-point extraction adapted to depend on the different and specific resolution levels in each FS, possibly differing from the sampling radius used $d_{0},\ldots ,d_{m}$. We also use the modified correspondence grouping algorithm that accommodate the extraction of the key-points in each point cloud of the multifoveation. The points of the scene are downsampling using several radii $r_{k}$ for each *k* level and k∈[0,…,m], where all the foveated structures of the scene have the same numbers of levels. From the arbitrary determination of the extreme radii of the structures are the first level (level 0) having a radii $r_{0}$ and last (level *m*) using a distance $r_{m}$. The intermediate levels use linear interpolation for radii estimation, similar to what is done in the size of the boxes of the foveated structures.

Therefore, by adopting these radius downsampling approaches made in [7], considerable time savings can be achieved by reducing the number of key-points both in the computing of descriptors and in the matching step, resulting in the large increase in the density of the key-points near the fovea position without significant increase in the total number of original points in the scene. Thus, it improves the efficiency of the detection of objects if the foveated structures are defined correctly and reduces the number of false matches of descriptors.

Notice that the outputs of our proposed scheme (Figure 4c) and of the classic scheme (Figure 4c) have some differences. The FPC model can only recognize several objects if all are close, considering a fovea box which does not cover much of the PC. In this new MFPC model, the objects can be identified without this restriction, since the distribution of multiple structures in the scene allow the proper positioning of each zone of maximum resolution.

## 6. Experiments

The compaction and performance of the 3D multifoveated point cloud model are evaluated in one experiment. In this proposal, the resources used are verified based on the mechanism, methodology used and the specific objectives of each experiment. All experiments were performed on a desktop with an Intel Core i7 2600-K 3.40 GHz processor and 8 GB of RAM.

The experiments were done aiming at comparing our mechanism with other models that use object recognition [35] as an integrated application, so that we can expose the capabilities and limitations of the proposed model. Next, a series of detailed exercises were carried out to observe the main differences between our approach that makes the treatment of redundancies and a raw multifoveation (without treatment redundancies). The limitations and open problems left by the proposed MFPC mechanism are presented.

### 6.1. Dataset Used in Experiments

**Figure 5:**
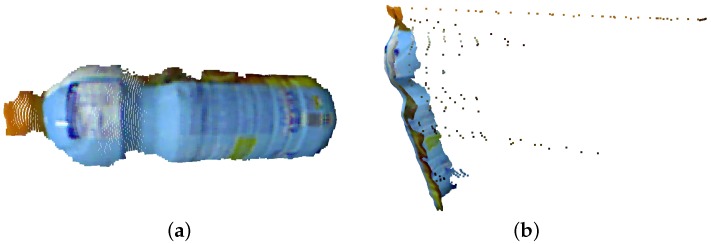
Illustration of the model used to perform object detection: (**a**) the front-end model; and (**b**) the side-mounted model. The PC is acquired from the works of Tombari et al. [51,52].

Through the datasets provided by Tombari et al. [51] and Tombari [52], we set up some situations to test the proposed system (MFPC) in relation to other models with object recognition. In this way, we used the object of interest shown in Figure 5. This model represents a bottle of juice in NP with a total of 9968 points. This dataset was used because it already has a ground truth to verify the results and obtain a better precision in the comparison between approaches.

**Figure 6:**
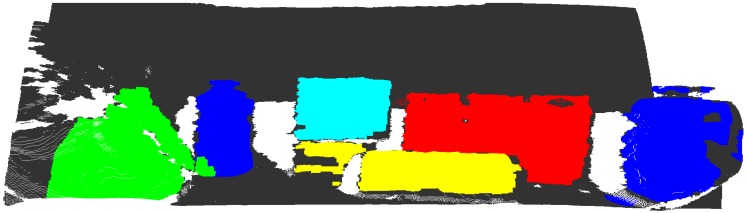
Representation of the dataset illustrating the scene with objects. It is shown the representation of ground truth where each type of object is highlighted in a different color (our target is blue). The PC was acquired from the works of Tombari et al. [51,52].

The scene and model used was the one provided by Tombari et al. [51]. Figure 6 show the representation of a table with some household products, where one of these products is a juice bottle (our model of Figure 5) arranged a little apart one from another, so the scene has a total of 281,097 points.

### 6.2. Tools for Methodology Validation

In this experiment, we analyzed the reduction of the number of points and key-points, the total number of matches performed, the mean time, the time estimate in relation to a confidence grating, the number of objects detected and the association of the identified features. According to the literature [7,8,30,33], we used this approach to evaluate the performance of the descriptors and foveated models.

The application used to justify the results of our proposal uses feature matching to perform the association with a object model. Thus, it is an instance classification problem, i.e., a feature association problem. According to Da Silva [8], the classification problem can have the following classifications for the associated features: true positive association that was established correctly, false positive association established incorrectly, true negative association not established correctly, and false negative association not established correctly. However, in this proposal, we do not have exclusive interest in the classification of the key-points by the descriptors, since these points were fully discussed in previous works [7,30,33] in relation to the 3D descriptors with focus in SHOT.

In this work, we use the confidence interval of 95% based on the t-Student distribution to indicate the reliability of the estimation of the times of the experiments performed, used for experiments with up to 30 samples, which is a small set of samples, for the estimated run times of each chosen approach the calculation was made from Equation (9). For this equation, we have *S* standard deviation, $\bar{x}$ sample mean, *n* sample number and $t=\phi^{-1}\left(0.975\right)=\phi^{-1}\left(0.025\right)=1.96$ the quantile of the t-Student distribution.

(9) $$\begin{array}{c} P\left(\bar{x}-t\frac{S}{\sqrt{n}}\leq \bar{x}\leq \bar{x}+t\frac{S}{\sqrt{n}}\right)=0.95 \end{array}$$

Since this is a key-point correspondences problem (local features), we complement our analysis using an approach assembling comparative graphs [8] that address and emphasize the precision and sensitivity of the chosen methods. This is done based on ground-truth (estimates considered true, i.e., a model to be followed) provided by [51] and Tombari [52]. The classification is made from the execution of Algorithm 4 that establishes a relation between the local features of the model and scene, returning the total amount of correspondences made. Algorithm 5 performs grouping by correspondence, which provides the instances for the estimation of object relationships, where these estimated instances can be classified as true-positive or false-positive. Then, to determine the accuracy of the approaches, we use Equation (10) and the sensitivity is calculated by Equation (11).

(10) $$\begin{array}{c} \mathrm{precision}=\frac{N^{\circ }\;\mathrm{correct}\;\mathrm{matches}\;(\mathrm{true}-\;\mathrm{positive})}{N^{\circ }\;\mathrm{total}\;\mathrm{matches}\;(\mathrm{true}\;\mathrm{and}\;\mathrm{false})} \end{array}$$

(11) $$\begin{array}{c} \mathrm{sensibility}=\frac{N^{\circ }\;\mathrm{correct}\;\mathrm{matches}\;(\mathrm{true}-\;\mathrm{positive})}{N^{\circ }\;\mathrm{total}\;\mathrm{matches}\;\mathrm{to}\;\mathrm{be}\;\mathrm{made}} \end{array}$$

To facilitate the understanding and presentation of the results, we list the six approaches in Table 2. This simplifies the layout of the approaches in the exposed graphs, seen later. As it is not a question of evaluating only the descriptor capacity, but also the ability of the models to perform the task of recognizing a model in a point cloud, we do not use the 1−precision×recall curve used by Silva and Gonçalves [8], Salti et al. [30], and Tombari et al. [33].

### 6.3. Non-Foveated Experiment

The application selected to check the capacity of our proposal has several types of parameters such as those shown in Table 1. Thus, before performing any type of experiment, it is necessary to tune the related parameters in relation to both object recognition and foveation. We work out the tuning based on the values already used previously [7,33,35] as starting points and we arrive at the values exposed in this section in an empirical way. The tuning has a focus on the detection of all objects in a correct way and in the shortest time, which is the applicable case of each selected approach, disregarding for the foveated cases the positioning mechanism of the foveae (i.e., when positioning is already stabilized).

**Figure 7:**
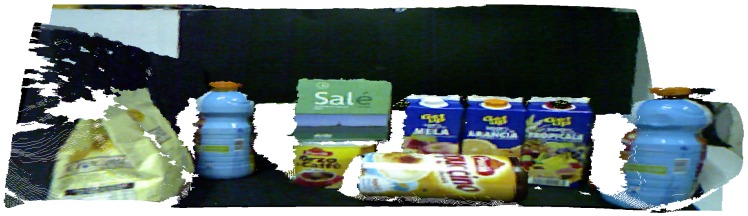
Representation of the dataset illustrating the scene with objects. The original point cloud used by [51] and Tombari [52] is shown in parallel projection view.

The standard approach of the non-foveated application, i.e., pure application of the proposal presented by Tombari and Stefano [35], is tuned to the parameters presented in Table 3 for the scene referring to Figure 7. We have selected this method to visualize the major differences between our proposal and the original application. In addition, the parameters in Table 3 are used in the other experiments that approach the scene of Figure 7.

### 6.4. Experiment with Foveation Covering All Object (FCAS)

**Figure 8:**
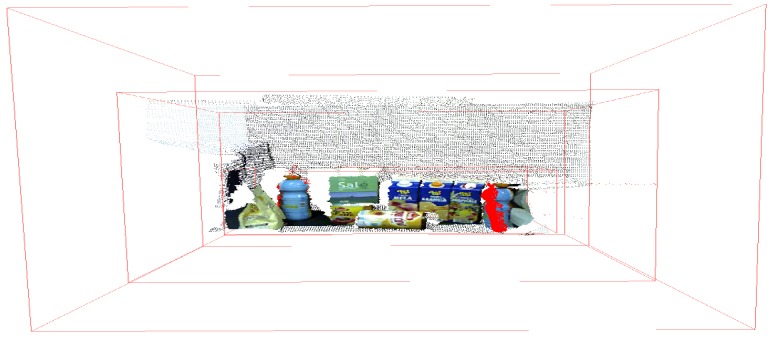
The model identified was a juice bottle illustrated in Figure 5a,b, where its parameters are extracted and highlighted in red. The results of the experiments related to Experiment 4 (FCAS).

Similar to the object recognition task, the integrated foveated models have several types of parameters to specify. However, some of them very simply perform the tuning, for example positioning the boxes, sizes and number of resolution levels. This would simplify the use of the model in some tasks as for example: tracking and tracing patterns. Thus, for the scene referring to Figure 8, we use a single foveated structure; consequently, it is the FPC model proposed by Beserra Gomes et al. [7].

The parameters defined for this approach allow encompassing the two desired objects in the fovea box; these parameters are exposed in Table 3 and Table 4. Table 3 has the additional parameters for object recognition. The purpose of this experiment is to observe the ability to identify multiple objects that are involved by a single fovea, where they have a relative distance between them, as shown in Figure 6.

### 6.5. Experiment with Foveation Covering One Object (FCRM/FCLM)

**Figure 9:**
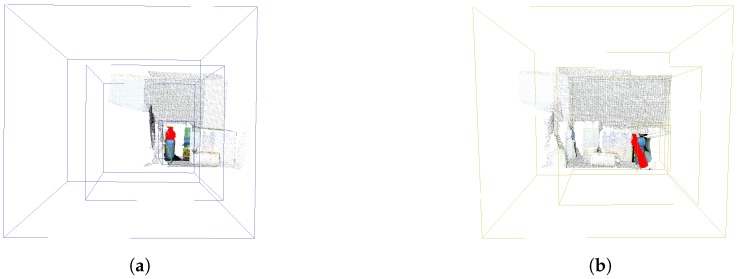
The identified model was a juice bottle illustrated by Figure 5a,b, where its parameters are extracted and highlighted in red. (**a**,**b**) The visual results of the experiments related to Experiments 6 (FCLM) and 5 (FCRM), respectively. The mapping of experiment numbers to their respective descriptions is shown in Table 2.

The strategy of covering one object at a time in the occurrence of several objects in the scene, using the FPC as suggested by Beserra Gomes et al. [7] is one of the approaches that we adopt here for the comparison. The parameters of this experiment are set out in Table 5 where the changes are in the positioning of the extreme boxes (smaller and bigger boxes) and the size of the smallest box, since the focus becomes on the objects individually. The purpose of this configuration is to deal with the simultaneous detection of objects when there is only one fovea structure. We disregard here the technique that will define the positioning of this single structure in simultaneous instants. Thus, the environment of this experiment is given in the situation where the detection of an object is made and immediately the fovea structure would be positioned on top of the next object, as shown in Figure 9a,b.

### 6.6. Multifoveated Experiment

**Figure 10:**
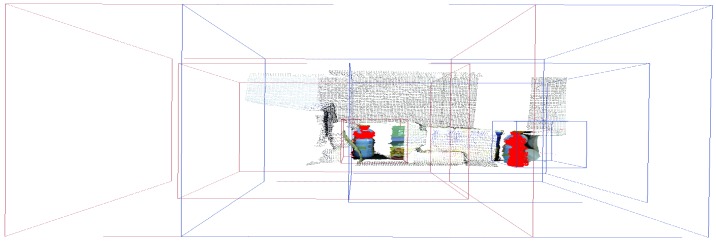
The identified model was a juice bottle illustrated by Figure 5a,b, where its parameters are extracted and highlighted in red. It is the visual result of the configurations related to Experiment 3 (MFPC) and it is noticeable that the two structures presented are the ones that make up Figure 9a,b.

Given these three scenarios chosen for analysis of the scene represented in Figure 7, we focus our attention on the multifoveated models. The theme of our proposal that is the multifoveated 3D point cloud model has the same line of the multifoveated raw approach so there would be no advantage to any mechanism. Basically, we use the configurations of the two structures that focus on each object (experiments 6 and 5, FCLM and FCRM, respectively) in the multifoveated context to try to achieve more equivalent results. The basic difference between the multifoveated models presented in this proposal is the treatment of the redundancies. They are caused by the intersections of the foveated structures which are made in Algorithm 1. Thus, the configurations of the multifoveated models are expressed in Table 6 and the visual results are shown in Figure 10.

## 7. Results

After deciding the types of strategies to choose for comparison, we set out to execute each of them to acquire information. Two groups of graphs are used to display the information acquisitions. They are made up of four types that join the six approaches providing a better comparison between the strategies.

The first group of graphs brings information that is related to the performance of the approaches: quantity of points, number of key-points, average time and number of objects found. The second group of graphs tries to explain the accuracy of the strategies in relation to the characteristics of the execution of the algorithm of object detection of Tombari and Stefano [35]. The characteristics of this group are: correspondence between points, amount of true-positive, precision and recall. For each scene, the two groups of graphs were used, besides elaborating tables which allow having a better precision in the execution times sampled of each scene for each strategy.

**Figure 11:**
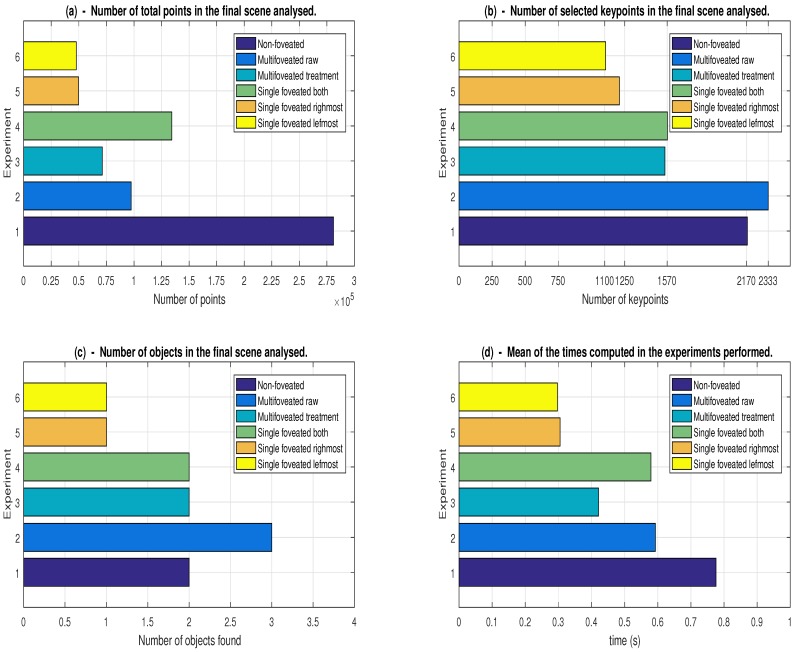
The result group of the strategies performances carried out referring to the scene illustrated in Figure 7: (**a**) the total number of points in the final scene analyzed; (**b**) the result of the number of selected points of key-points in the final scene analyzed after execution of the strategies; (**c**) the amount of objects recognized for each configuration presented; and (**d**) the average of the times computed in each experiment performed where this result can be noticed in Table 7 (see more details in the text). The matches of the experiment numbers can be seen in Table 2.

**Figure 12:**
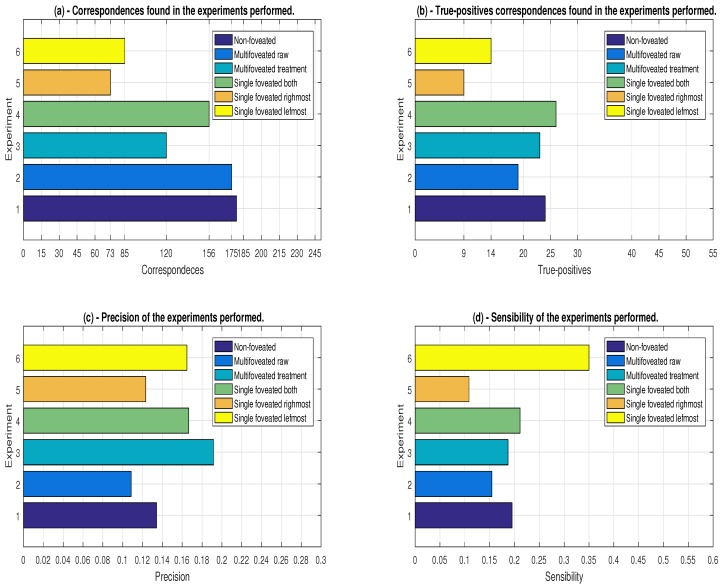
The result group of the strategies accuracies carried out referring to the scene illustrated in Figure 7: (**a**) the total number of matches performed in the final scene analyzed; (**b**) the result of the number of true-positive selected in the scene; (**c**) the precision of each strategy calculated from Equation (10); and (**d**) the sensitivity of each strategy using Equation (11) (see more details in the text). The numbers of the experiments are mapped in Table 2.

The related groups are shown in Figure 11 and Figure 12, where we have the performance group and the accuracy group, respectively. The performance of our proposal was one of the concerns, since the recognition algorithm requires many computational resources in relation to 2D proposals. In the set of graphs referring to Figure 11, the ordinates axis is related to the strategies used mapped in Table 2.

### 7.1. Results Analyzed from Performance Group

When analyzing the data shown in Figure 11a, we can see that the number of points (abscissa axis) of the proposition non-foveated (experiment 1) is much more accentuated than the models with single fovea and multifoveated. The model non-foveated has the same total of points of the original scene that is 281,097 points, since the models that use the foveation are with quantities below 150,000 points. This is because downsampling is executed at the levels of the foveated models, that is, decreasing the resolution as the levels move away from the fovea box. In our proposal (Experiment 3 (MFPC), we noticed that it is the third largest decrease of points, losing only to the experiments that are involving only one object, which are the FCRM and FCLM. The single foveation involving the two objects at the same time, Experiment 4 (FCAS), has the smallest reduction of points between the foveated models, This is because the objects are more distributed throughout the scene and, consequently, generate a fovea box with greater volume in order to involve them, in relation to the other foveated experiments for this scene.

The multi-foveated raw model (Experiment 2) has a convenient reduction of points even generating redundancy of points, a fact that is explained by the arrangement of structures where only the lower density levels have intersections. An expected fact is that the sum of the point quantities of the FCRM and FCLM strategies is equal to the amount of the raw strategy, so that 49,679 plus 47,776 equals 97,455, values of the respective experiments cited.

In addition to the amount of redundant points of Experiment 2 is equivalent to the difference between raw and MFPC strategies, that is, the amount of 18,573 points. Then, it is shown in Figure 11a that our strategy would have advantage over the others in the amount of points used to find all objects, using fewer points for the scene with more distributed objects.

Looking at the result graphs of extraction of the key-points based on each strategy (Figure 11b), we can see that the number of points in the scene is not proportional to the extraction of key-points. This extraction depends on the positioning of the fovea boxes for the foveated models. Note that Experiment 4 with a much higher number of points compared to Experiment 3 has a similar amount of extracted key-points, i.e., it is possible to preserve the descriptiveness of the scene with a significant reduction in the number of points.

In the strategy that dedicates a foveated structure to serial processing (Experiements 5 and 6), we can see that the number of key-points is inferior in relation to the other experiments, because they focus on only one object at a time, but when joined together they are approximately the same portion of Experiment 2. That is, much more features are verified to analyze each object serially than together. In the multifoveated raw, more key-points are found than in the non-foveated model. These key-points are duplicated or distorted in the scene generated by the redundancies, what affects the local descriptiveness by modifying it, being this the main problem of the approach multifoveated raw.

The expectation of the objects found is represented in the graph shown in Figure 11c. We can see that all strategies have expected results, except the strategy that uses the multifoveated raw. This fact can be explained initially using Figure 11b, since we have a higher number of key-points in relation to the non-foveated strategy with the same configurations. The scene distortion caused by the multifoveated raw has brought this false detection. This actually shows at first that this type of strategy is not reliable, even for few used foveated structures.

The performances of the execution times of the strategies used are illustrated in Figure 11d. We can see that the time of the experiment without foveation is much superior compared to the strategies taken. The graph of performances of the execution times of Figure 11d strategies is constructed with 15 consecutive samples of execution times of each strategy, which allows to elaborate a confidence interval that guarantee a good estimation of the average, as seen in the Table 7. As previously mentioned, Experiment 6 and 5 represent the strategy of using only one foveated structure to identify several objects at a time. The evaluations of the average times of performance of these two experiments are combined for comparison with the shortest average time (experiment MFPC) obtained in Table 7 reduction column. Table 7 has the following information: $I_{lower}$ (lower end), $I_{upper}$ (upper end), μ (arithmetic mean), $\sigma^{2}$ (variance) and reduction of time in relation to the experiment MFPC. The ends are maximum and minimum fluctuation of the acquired times of each experiment done.

We can see in Figure 11d that the best performance in the identification of all objects, given the configurations already shown, is the multifoveated approach with redundancy treatment (our proposal). This is expected, since we have one of the largest points reduction relative to the original scene, as shown in Figure 11a. Table 7 presents the mean of the execution times in a more precise way, the maximum and minimum confidence intervals, the variances of the experiments and the reduction ratio in relation to our MFPC model to obtain a clearer analysis. Comparatively, the multifoveation with treatment (MFPC) has more than 25% of time reduction than the other strategies used. We consider that all objects should be identified in the scene. It is then observed that this performance makes our MFPC a good alternative for environments that have similar object layouts as the one shown in Figure 7.

Then, for the group of performance graphs (Figure 11), it is noted that the ability to reduce the number of points around the desired objects allows a reduction in execution time. However, it does not proportionately reduce the scene’s descriptiveness, one of the positive points of the methods taken. It is also possible to observe that the amount of key-points is not necessarily a sign of improvement in the quantity and quality of the features as the multifoveated raw has more selected key-points than the original scene and, even then, there is the recognition of an object that does not exist in the scene. This fact occurred due to the redundancies generated, as already explained.

### 7.2. Results Analyzed from the Accuracy Group

Given these circumstances, we have drawn up charts to deal with the accuracy of the strategies (Figure 12). Figure 12a shows the matches that are made. That means the total number of selected points that could represent some point of the model object. It can be noticed that the approaches non-foveated, raw and FCAS are those that have a greater number of correspondences. As already expected, Experiment 1 (non-foveated) is the one that has the largest number, as it is the one which has not undergone downsampling. Experiment 4 (FCAS) has this high number of matches because of the size of the fovea that covers highly descriptive areas which have high resemblance to the model. The multifoveated raw Experiment 2 has the most descriptive and similar regions between the model and the scene in its fovea boxes. Unlike the redundancy treatment strategy, this experiment duplicates the space-changing points and distorts the scene bringing more false correspondences than the strategy with treatment, being able to be proven by the result in Figure 11c. The MFPC, FCRM and FCLM experiments have their respective expected match results, since they were more directed to the desired objects, as shown in Figure 9a,b and Figure 10.

Analyzing the results by the number of valid hits, that is, the number of true-positive, we construct the graph represented in Figure 12b. An interesting fact in this analysis is the result obtained with the single fovea model that covers all the objects. It obtained the highest value of true-positive surpassing the non-foveated strategy. This fact can be explained by the configuration focus in the areas with high concentration of descriptiveness and similarity with the desired objects. We can also notice that Experiments 1, 3, and 4 have similar results, as shown in Figure 12b. Practically, Experiments 1 (non-foveated) and 3 (MFPC) have the same true-positive numbers, showing that our proposal does not cause a large change in the validity of the data found, whereas the multifoveated raw model has a minimal advantage in the amount of valid matches compared to the Experiment 3, even getting much more matches and more points. It is observed that the duplication of points, which could be considered as reinforcement in the 3D environment, distorts the representation and the points’ descriptiveness for the object recognition algorithm proposed by Tombari and Stefano [35].

The precision and sensitivity graphs are shown in Figure 12c,d, respectively. Precision and sensitivity calculations are made based on Equations (10) and (11), respectively. We observe that the foveated models usually maintain the precision and sensitivity as seen in the presented graphs. They can have superior performance of precision in relation to the model no-foveated, as seen in Figure 12c, which shows Experiment 3 with precision close to 20% and the no n-foveated approach (Experiment 1) with accuracy <15%. In relation to the sensitivity, it is the fraction of the relevant points identified. Given that the configuration for the extraction of key-points are the same for all strategies, we have the leftmost object with 40 in total to be identified, while the rightmost object has 83 key-points. This led to a higher sensitivity in the single fovea strategy that is positioned on the leftmost object, as shown in Figure 12d, since the difference is not so great among the true-positives in each strategy. Based on the presented sensitivity results, the foveated models have similar results in comparison to the original model (non-foveated), except for the multifoveated raw model, and the highest percentage is the FCAS (4) among the foveated approaches. Thus, for tunings and the scene chosen, we have seen that the proposed multifoveated brings improvements in the reduction of quantity of points, execution time and precision in relation to Strategies 1 and 4, in addition to noting that the multifoveated raw is not feasible for the various problems mentioned.

## 8. Discussion and Limitations

The results presented make it clear that the multifoveated model has advantages over other strategies when there is adequate treatment of the redundancies generated by the intersections between structures, since it manages to preserve the correct number of correspondences of the original strategy [35] and has better accuracy and execution time in relation to other strategies. It is shown that the multifoveated strategy without redundancy treatment is impracticable if there is no guarantee that there will never be an intersection between the FS, since overlapping performance was lower than our proposal. We also found that the problem related to object recognition when it is arranged in two levels of a foveated structure of the FPC model was solved, showing results and configurations of some provisions of the fovea boxes.

However, we must consider that our proposal needs a distribution approach of the foveated structures. Thus, the results presented in the object comparisons occur when the object has already been found, although the disposition strategy of the foveated structures can be applied in parallel with our proposal.

Our mechanism is aimed at working with generic point clouds without any organization. Thus, when we build each level of the foveated structures, we pass through all the points of the original cloud, causing an inefficiency of the proposal at this point. Thus, we see that a pre-organization in the PC is further necessary for an optimization in the proposed model.

The tuning of the algorithm parameters and the tasks are a complex effort to achieve, since it is possible to manipulate the point clouds with the possible objects with different densities. For example, consider an object within the level *k* and another object in k−1. This brings some obstacles in the tuning of the method along with the parameters of the expected task. However, notice that, as already mentioned, either the FPC model or the MFPC model proposed in this work can be used in other types of tasks such as 3D data compression, tracking objects and visual attention control tasks.

## 9. Conclusions

This work has proposed an approach for reducing the amount of point cloud data captured by RGB-D sensors using multiple structures borrowed from the work of [7]. This mechanism has been integrated and tested in the object recognition task as exposed by Tombari and Stefano [35] and can be integrated into tasks involving visual attention control and recognition. The proposed mechanism provides a considerable reduction of execution time in relation to other types of approaches between models and the original approach.

This article has three main contributions. The first one is the proposed mechanism that uses several foveated structures that can be found without producing redundant points in a PC. Untreated occurrences would cause serious problems in the integrated tasks. The second is that the work provides the conservation of the density hierarchy of the levels of the structures, that is, the lower density levels are superposed by the larger ones without occurrences of redundant points. The last contribution relies on the investigation of the problems related to FPC [7]. That investigation allowed noticing that the source of the problem at that scheme is caused by the descriptor used in the proposal, SHOT [33], which has sensitivity to the variation of the point density. This is solved with the improvements made by Salti [30] that greatly reduced the sensitivity, making it possible to perform the task without occurrence of detection problems.

Depending on the task, foveated structures should be positioned appropriately, together with their respective fovea boxes that are guided by their respective fovea vectors so that the task can be successfully performed. However, a consensus has not been reached on how to elaborate this strategy in the 3D space, where one of the main challenges of the models studied is how the fovea should be positioned. For our approach, the challenge is bigger, since we deal with more than one foveated structure, so we need a logistics of how to initially position each of the structures. A uniform distribution seems to be a good strategy, which can be used for some tasks as object detection.

The ability to use multiple point cloud attention focuses allows more complex tasks to be operated in parallel. In this sense, the proposed mechanism enhances the previous proposal [7]. We could reproduce results with a greater reduction in the number of points in the context of multiple objects in the scene compared to the predecessor in possible applicable configurations and, consequently, the results of the time execution are better. In this way, the proposal is more effective, as could be verified on the results of the realized experiments, approaching the use of the task of object recognition in applications that require real-time response. However, we still have the problem of how to perform the positioning of foveated structures, which should be studied. A possible strategy could be the use of a hybrid model, that is, the use of image acquisition to define how to position the structures foveated in the given 3D scene as 2D models have a lower response time than 3D ones.

## Figures and Tables

**Table 1:** List of parameters for the movement of foveated structures and their respective default values. All parameters are provided in meters except for $m_{f}$ is quantity.

Param	Description	Default Value
$m_{f}$	Resolution (levels-1) number	3
$S_{0,f}$	Smaller density box size	(3.0,3.0,3.0)
$S_{m,f}$	Larger density box size	(0.5,0.5,0.5)
$F_{f}$	Position of fovea box	(−0.07,0.02,0.6)
$\Delta_{f}$	Position of the outermost box	(−2.9,−1.9,−1.3)
$r_{0,f}$	Smaller radius key-point size	0.08
$r_{m,f}$	Larger radius key-point size	0.02

**Table 2:** Enumeration of the treated approaches in the comparison of results for both the scenes the comparison experiments.

N°	Description
1	Non-foveated [35]
2	Multifoveated *raw*
3	Multifoveated with redundancy treatment (MFPC)
4	Foveated covering all object simultaneously (FCAS)
5	Foveated covering the rightmost object (FCRM)
6	Foveated covering the leftmost object (FCLM)

**Table 3:** List of parameters of the matching algorithm and their default values (see text in more detail). All parameters are provided in the scene in the unit of (meters).

Parameters	Description	Default Value
$k_{ns}$	K-Neighbors normals scene	10
$r_{ks}$	Radius key-points scene	0.03
$r_{ss}$	Radius SHOT scene	0.035
$r_{ls}$	Radius LRF scene	0.015
$k_{nm}$	K-Neighbors normals model	10
$r_{km}$	Radius key-point model	0.01
$r_{sm}$	Radius do SHOT no model	0.02
$r_{lm}$	Radius do LRF no model	0.015
$d_{max}^{2}$	Correspondence threshold	0.25
$L_{h}$	Size of Hough cell	0.04
$V_{h}$	Voting threshold	3.0

**Table 4:** List of parameters of the approach with 3D moving fovea and its standard values (see text in more detail) for the experiment concerning Figure 6. All parameters are provided in the scene in the unit of (meters).

Parameters	Description	Default Value
*n*	Number of foveated structures	1
*m*	Number of resolution levels - 1	3
$S_{0}$	Smaller density box size	(1.5,1.5,1.5)
$S_{m}$	Larger density box size	(0.7,0.3,0.4)
Δ	Position of the outermost box	(0.05,0.1,0.75)
*F*	Position of fovea box	(−0.7,−0.8,0.5)
$r_{0}$	Smaller radius key-point size	0.05
$r_{m}$	Larger radius key-point size	0.03

**Table 5:** List of match parameters by unique foveated structures for each object in the scene (experiment in Figure 6). The other parameters are according to Table 4.

Fovea	$S_{m}$	Δ	*F*
Right	(0.2,0.3,0.3)	(0.35,0.1,0.5)	(−0.7,−0.8,0.5)
Left	(0.2,0.3,0.3)	(−0.15,0.09,0.75)	(−1.2,−0.8,0.5)

**Table 6:** List of multifoveated matching parameters for the involvement of the two objects in the scene (experiment in Figure 6). The other parameters are according to Table 4.

*n*	*m*	$r_{0}$	$r_{m}$	$S_{0}$
2	3	0.05	0.03	(1.5,1.5,1.5)
	**Fóvea**	$S_{m}$	F	Δ
	1	(0.2,0.3,0.3)	(0.35,0.1,0.5)	(−0.7,−0.8,0.5)
	2	(0.2,0.3,0.3)	(−0.15,0.09,0.75)	(−1.2,−0.8,0.5)

**Table 7:** Comparison of the execution times of the strategies performed for the scene referring to Figure 7. The confidence interval used is 95% by t-Student.

N°	$I_{\mathrm{lower}}$ (s)	μ (s)	$I_{\mathrm{upper}}$ (s)	$\sigma^{2}\left(s\right)$	Reduction by Exp. 3%
1	0.7649	0.7760	0.7871	$4.040\times 10^{-4}$	45.73
2	0.5817	0.5928	0.6039	$4.015\times 10^{-4}$	28.96
3	0.4148	0.4211	0.4273	$1.271\times 10^{-4}$	0
4	0.5644	0.5785	0.5927	$6.530\times 10^{-4}$	27.21
5	0.3016	0.3046	0.3076	$0.256\times 10^{-4}$	-
6	0.2940	0.2968	0.2996	$0.291\times 10^{-4}$	-
6+5	-	0.5956	-	-	29.98
